# Clinical Aspects and Prognosis Evaluation of Cirrhotic Patients Hospitalized with Acute Kidney Injury

**DOI:** 10.1155/2019/6567850

**Published:** 2019-03-03

**Authors:** Célio Geraldo de Oliveira Gomes, Marcus Vinicius Melo de Andrade, Ludmila Resende Guedes, Henrique Carvalho Rocha, Roberto Gardone Guimarães, Fernando Antônio Castro Carvalho, Eduardo Garcia Vilela

**Affiliations:** ^1^Graduate Program in Science Applied to Adult Health, Medical School, Federal University of Minas Gerais (Programa de Pós-Graduação em Ciências Aplicadas à Saúde do Adulto, Faculdade de Medicina da Universidade Federal de Minas Gerais), Brazil; ^2^Alfa Institute of Gastroenterology of the Clinical Hospital of the Federal University of Minas Gerais (Instituto Alfa de Gastroenterologia, Hospital das Clínicas da Universidade Federal de Minas Gerais), Brazil

## Abstract

**Background:**

Acute kidney injury occurs in approximately 20% of hospitalized cirrhotic patients. Mortality is estimated at 60% within a month and 65% within a year.

**Aims:**

To evaluate survival in 30 days and in 3 months of cirrhotic patients hospitalized with acute kidney injury, identifying factors associated with mortality.

**Methods:**

52 patients with cirrhosis admitted to an academic tertiary center who presented acute kidney injury according to the International Club of Ascites criteria were evaluated. Clinical and laboratory data was collected at diagnosis between 2011 and 2015.

**Results:**

Average age was 54.6 (±10.7) years and 69.2% were male. The average MELD, MELD-Na, and Child-Pugh scores were 21.9 (±7.0), 24.5 (±6.7), and 10.1 (±2.2), respectively. Thirty patients (57.7%) were in acute kidney injury stage 1, 16 (30.8%) in stage 2, and six (11.6%) in stage 3. Mortality was 28.6% in 30 days and 44.9% in three months. In multivariate analysis, variables that were associated independently to mortality were lack of response to expansion treatment and Child-Pugh score. Mortality was 93.3% in three months among nonresponders compared to 28.6% among those who responded to volume expansion (p<0.0001).

**Conclusion:**

Acute kidney injury in cirrhosis has dire prognosis, particularly in patients with advanced cirrhosis and in nonresponders to volume expansion.

## 1. Introduction

Acute kidney injury (AKI), a frequent complication of liver cirrhosis, is present in approximately 20% of hospitalized patients with cirrhosis [1]. Estimated mortality of such patients is of around 50% in a month and 65% within a year [2]. AKI is also implicated in worse prognosis for patients with cirrhosis admitted in intensive care units [3] and outpatient clinics [4] and is associated with reduced survival following liver transplants when compared to patients without AKI [5, 6]. When associated with other complications, such as infections, digestive bleeding, and alcoholic hepatitis, AKI also contributes to worse prognosis [7–9]. Data regarding prevalence and prognosis of AKI are scarce in Brazil [10].

The goal of this study is to evaluate factors associated with prognosis of AKI in patients with cirrhosis admitted to the Clinical Hospital of the Federal University of Minas Gerais (UFMG).

## 2. Material and Methods

Patients over the age of 18, admitted to the Clinical Hospital of UFMG with a diagnosis of AKI upon admission or any time during their hospital stay, were included in the prospective observational study. The exclusion criteria were previous dialysis treatment, previous kidney or liver transplant, and malignant tumors (with the exception of hepatocellular carcinoma).

Cirrhosis diagnosis was based on liver biopsy, when available, or a combination of clinical, laboratory, endoscopy, and imaging findings that were compatible to the disease.

AKI was defined according to the criteria proposed by the Acute Kidney Injury Network (AKIN) [11], revised by the International Club of Ascites (ICA) [12] (Table 1). Stage 1 was subdivided into 1a and 1b, taking into consideration creatinine values of 1.5mg/dL or less and higher than 1.5 mg/dL, respectively, as proposed by Fagundes et al. [13]. The presence of two creatinine values with a difference of at least 0.3mg/dL in 48h or an increase of at least 50% in baseline value was used for AKI diagnosis. Baseline creatinine was defined as the most recent and stable value, up to three months prior to hospital admission. For patients without a baseline stable serum creatinine in the last three months, serum creatinine at admission (or closest to admission) was used as the baseline value. Therefore, although the study began in 2011, the diagnostic criteria for AKI were basal creatinine value according to the most recent ICA consensus [12].

Patients were etiologically divided into six groups: those with infection (spontaneous bacterial peritonitis was defined by presence of at least 250 polymorphous leucocytes per mm^3^ in analysis of ascites liquid in the absence of a source of infection in peritoneal cavity; spontaneous bacteremia was defined as positive blood cultures in absence of infection site; pneumonia, urinary tract infection, and skin and soft tissues infection were defined according to established clinical practice diagnostic criteria); hypovolemia (when patients had a history of loss of body fluids in days prior to diagnosis, such as use of higher doses of diuretics, relief paracentesis, and digestive tract hemorrhage), in the absence of other possible causes, associated with at least one of the following: increase in rate between blood levels of urea and creatinine to values higher than 40, urinary sodium below 10mEq/L, sodium urinary excretion below 1%, and excretion of urea fraction below 35%); parenchymatous nephropathy, defined by protein/creatinine ration of over 0.3 or abnormal urinary sediment with over 50 red blood cells per field, or abnormal findings in renal ultrasound; hepatorenal syndrome, defined according to ICA 2007 criteria [14] (these criteria were used instead of more recent criteria in study group [12] because the latter was published when most patients had already been included in this study); miscellaneous, when the cause of AKI did not fit in any of the previous categories, and multiple when more than one cause of AKI was identified.

Once a possible AKI case was identified, patients were submitted to careful review of clinical history, complete physical examination, blood samples drawn (blood cell count, sodium, potassium, chlorine, magnesium, glucose, liver enzymes, albumin, prothrombin activity and INR, partially activated thromboplastin time, urinary sodium, urinary creatinine, urinary urea, and urine analysis, as well as cultures of blood, urine, and study of ascitic liquid, when present, through cell count with differential leucocyte count, measurement of lactate dehydrogenase, albumin, total proteins, and culture in blood culture balloon) and total abdominal ultrasound in order to establish etiology.

Management of AKI followed the protocol used in the Gastroenterology Service of the Clinical Hospital of the Federal University of Minas Gerais, which included the following measures: (1) there was suspension of medication that could be inducive to AKI, particularly diuretics; (2) there was volume expansion according to degree of renal disfunction (patients with ICA-AKI 1 were given sodium chloride solution at 0.9% in a dose of 40 to 60ml/kg per day or human albumin at 20% at 0.5g/kg/day, for 48 hours; patients with ICA-AKI 2 and 3 were given human albumin at 20% in a dose of 1g/kg/day also for 48 hours or concentrated red blood cells if hemoglobin was below 7 or 8g/dL, in cases of digestive hemorrhage); (3) in the presence of infection, antibiotics were administered; (4) if spontaneous bacterial peritonitis was diagnosed, patients received human albumin at 20% at higher doses (1.5g/kg on day of diagnosis and 1.0g/kg 48 hours later); (5) when type 1 hepatorenal syndrome was diagnosed, patients received human albumin at 20 to 30 grams/day as well as terlipressin or noradrenaline.

During hospitalization, demographic, clinical, and laboratory data were collected to investigate predictors of mortality. Demographic variables included age and gender; clinical variables included Child-Pugh score, Model of End-Stage Liver Disease (MELD), MELD sodium (MELD-Na), ICA-AKI stage, response to treatment, and presence of ascites and hepatic encephalopathy. Laboratory variables were creatinine, albumin, total bilirubin, INR, C-reactive protein, and sodium from blood drawn at AKI diagnosis.

Primary outcome was mortality measured at 30 days and at 3 months.

The study was approved by the Ethics Committee at the Federal University of Minas Gerais and patients who chose to participate signed the informed consent form.

### 2.1. Sample and Statistical Analysis

Statistical analysis was performed using the program SPSS for Windows 17.0 (SSP Inc., Chicago, IL). Number variables were evaluated for normality through Kolmogorov-Smirnov test for selection of data presentation. Categoric variables were presented as percentages. Comparison was performed through Student's t-test or Mann–Whitney test (according to data distribution) and Qui-square (or Fisher test when appropriate). Univariate analysis was done to determine factors associated with mortality in 30 days and in three months. Variables with p<0.2 obtained through univariate analysis were included in Cox regression analysis. The adjustment of Cox regressive model was assessed by the Deviance test (p>0.05). Significance level was established at 5%. Survival rate curves were obtained through Kaplan-Meier analysis.

## 3. Results

Between October of 2011 and September 2015, a total of 52 patients were selected. Three were excluded from survival analysis since they received liver transplants within 30 days during follow-up. Therefore, 49 patients participated in survival analysis in the one-month mark. One discontinued follow-up after one month and, therefore, was not included in the three-month survival analysis. Patients' characteristics are displayed in Table 2. Average age was 54.6 (±10.8) years and 69.2% were male. The average MELD, MELD-Na, and Child-Pugh scores were 21.7 (±7.2), 24.4 (±6.9), and 10.1 (±2.2), respectively. Among causes of cirrhosis of the liver, alcoholic was the most common (30.8%), followed by viral (hepatitis B and C with 9.6% and 15.4%, respectively) and multiple causes (9.6%). Cryptogenic cirrhosis corresponded to 23.1% of cases. Ascites and encephalopathy were present in 86.5% and 28.8%, respectively.

Of the 52 patients, 30 (57.7%) were in stage 1 according to ICA-AKI, 16 (30.8%) in stage 2, and six (11.5%) in stage 3. Among the causes of AKI, infections accounted for 22 (42.3%) cases, hypovolemia for 15 (28.8%), hepatorenal syndrome for five (9.6%), multiple causes for four (7.7%), and parenchyma nephropathy for three (5.8%). Spontaneous bacterial peritonitis was the most common cause of infection, accounting for nine cases (40.9%); urinary tract infection and pneumonia accounted for three cases each (13.6%); skin/soft tissue infections, spontaneous bacteremia, and sepsis without focus had two cases each (9%). Hypovolemia was most commonly due to diuretics (46.6%) and digestive hemorrhage (40%). In the case of multiple causes, all cases had previous nephropathy diagnosis and developed AKI during an infection or use of diuretics. In the miscellaneous group, there was one case of acute pancreatitis, one of alcoholic hepatitis, and one of acute inflammatory abdomen.

The clinical and laboratory variables of survivors and nonsurvivors were compared at 30 days and at 3 months (Tables 3 and 4). The two groups were also compared regarding their response to treatment, as defined by ICA (Table 1). Patients who were alive at the end of the first month had lower Child-Pugh, MELD, and MELD-Na scores as well as lower levels of bilirubin and INR when compared to nonsurvivors (9.9 versus 11.3, p=0.019; 20.4 versus 25.1, p = 0.034; 23.1 versus 27.6, p=0.037; 1.72mg/dL versus 6mg/dL, p=0.028; 1.5 versus 2, p=0.012, respectively). Patients who responded to treatment also differed from the nonresponsive patients (p=0.001). At the end of the third month the same variables, except for INR, and including response to treatment, were lower in the patients that survived (p<0.0001). The levels of creatinine did not differ significantly between survivors and nonsurvivors in three months (2.0mg/dL vs. 2.2mg/dL, p=0.52).

**Figure 1:**
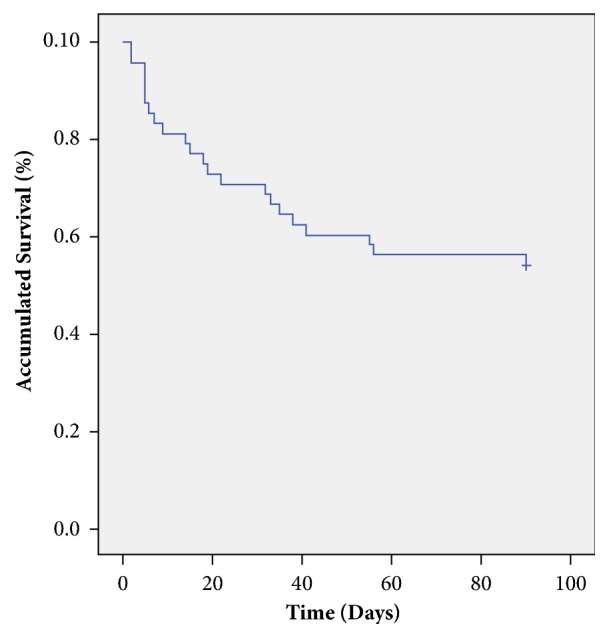
Survival in three months of cirrhotic patients admitted to hospital with Acute kidney injury (n=49).

As for patient survival, mortality was of 28.6% in 30 days and 44.9% in three months (Figure 1). Through Kaplan-Meier analysis based on ICA-AKI classification, of the 48 patients in which survival analysis was possible, those in ICA-AKI stage 2 presented higher tendency for greater mortality when compared to stage 1 patients (37.5% vs. 62.5%, p = 0.10). At the end of the third month there was no difference in survival rate between different stages (p=0.21). When stage 1 survival was compared to stages 2 and 3, no significant statistic difference was observed (p=0.194). Furthermore, no evidence of significant statistic difference for mortality was found in three months for patients with AKI in the two substages (28.6% vs. 40%, respectively, p=0.61).

For analysis of mortality predictors, Cox regression model was used. In the multivariate analysis for mortality evaluation in 30 days, variables with p lower than 0.2 were included: Child-Pugh, MELD, MELD-Na, total bilirubin, INR, and response to treatment. For the second analysis, following three months follow-up, the same variables were included, as well as the cause of AKI variable. Results are shown in Table 5. After statistical analysis, the variables that remained associated with mortality were response to treatment and Child-Pugh score, for both regression models. As for the first, those with response to volume expansion treatment showed a 74% reduction in mortality within a month and 75% in three months. Mortality of patients who did not respond to volume treatment was 92.3% (12/13 patients) and 28.6% in responders (partial or complete response) (10/35 patients) (p<0.0001). Survival was similar between partial and complete responders. As for the Child-Pugh variable, for every one-point score increase, mortality increased 63% in 30 days and 49% in three months.

## 4. Discussion

In concurrence with other reports in the literature, this study found evidence that cirrhotic patients with AKI have reserved short- and medium-term prognosis. Approximately one-third and 45% of patients deceased in 30 days and 3 months, respectively, following diagnosis. Belcher et al. [15], in prospective multicentric study involving 192 cirrhotic patients admitted to hospital care with AKI, diagnosed through AKIN criteria, reported 26% intrahospital mortality. Survival was correlated to AKI stage at diagnosis and was lower for more advanced AKI stages. Furthermore, AKI stage progression was an independent factor in association to mortality. Similarly, Scott et al. [16], in a cohort of 162 cirrhotic patients, 110 diagnosed with AKI and 52 controls, reported intrahospital mortality of 31.8%, versus 3.8% in control group (p<0.001). Mortality was proportional to AKI stage at diagnosis (13.5% stage 1, 37.8% stage 2, and 43.2% stage 3; p<0.001) and, in multivariate analysis, AKI was also an independent factor associated with mortality. Wong et al. [17] prospectively evaluated 166 infected cirrhotic patients with AKI and observed a 34% mortality rate in 30 days. Another prospective study involved 120 patients with cirrhosis and AKI [18], also based on AKIN classification, and their mortality rate was 46% in three months, a similar rate to our own findings.

Although mortality of cirrhotic patients with AKI remains high, an improvement in prognosis has been observed for these patients, particularly short term. In an extensive systematic review, including over eight thousand cirrhotic patients with renal dysfunction from 74 studies conducted between 1977 and 2010 [2], mortality in one month and three months was 58% and 71%, respectively, higher than the rates found in this study and in other recent studies that evaluated AKI prognosis in cirrhotic patients [15–18].

ICA's criteria for renal dysfunction in cirrhosis contributed to more efficient and uniform diagnosis and their stratification in stages made it possible to detect more severe cases. This may have contributed to reduction of mortality in cirrhotic patients with AKI. Of the 48 patients for which survival analysis was possible, those that fit criteria for ICA-AKI stage 2 tended towards higher mortality rates when compared to stage 1 patients (37.5% vs. 62.5%, p=0.10). However, there was no evidence of such tendency between stages 2 and 3 (62.5% vs. 40%, p=0.307). This might be explained by the reduced number of cases in stage 3 (n=5). Another explanation for this finding is that two of the five patients with stage 3 AKI, 40% of the cases, had either AKI following parenchymal nephropathy or multiple-cause AKI. According to Martin-Llahi et al. [19], parenchymal nephropathy has a better prognosis than other causes of AKI, such as infections, hypovolemia, and hepatorenal syndrome. This was supported by our findings. Mortality in three months for AKI associated with hepatorenal syndrome, infection, and hypovolemia was 100%, 50%, and 40%, respectively. There were no deaths in the group of patients diagnosed with AKI associated with nephropathy.

Although creatinine over 1.5mg/dL is associated with higher chance of AKI progression, as well as higher rates of mortality in IRA stage 1 patients, which suggests it may be advisable to subdivide them into stage 1a and 1b [13, 20], our findings did not show evidence of significant statistical difference in mortality at three months among patients with AKI in the two substages (28.6% vs. 40%, respectively, p=0.61).

Based on literature data, the variables most associated with prognosis of cirrhotic patients with AKI in univariate studies are age, encephalopathy, Child-Pugh score, total bilirubin, prothrombin time, sodium (blood and urine), and response to terlipressin [2]. In our study, besides Child-Pugh score, bilirubin, INR, MELD and MELD-Na scores, and response to treatment were associated with mortality.

On the other hand, in studies with multivariate analysis, Child-Pugh and MELD scores and their components, such as albumin, bilirubin, prothrombin time, encephalopathy, and age, were the variables most related to prognosis [2, 21]. In this study, Child-Pugh score and, above all, response to treatment were independently associated with mortality in 30 days and in 3 months. In fact, mortality of those who did not respond to volume expansion was 92.3% (12/13 patients), while only 28.6% of responders (partial or complete) died (10/35 patients) (p<0.0001). Survival was similar among those who had partial and complete response. Belcher et al. [15] report that reduction in blood creatinine levels was significantly more common in survivors when compared to nonsurvivors (42% and 22%, respectively; p=0.01). Conversely, in cirrhotic patients that progressed in AKI stage, mortality reached 70%. Wong et al. [17] reported mortality of 80% in those that did not recover renal function, higher than patients with partial (40%) or complete (15%) response to treatment with albumin, associated or not to midodrine and octreotide (p<0.0001). These findings are compatible to the latest ICA consensus [12], which proposes early use of vasoconstrictors for cirrhotic patients with AKI that do not respond to treatment with crystalloids or albumin, particularly stage 1 AKI with creatinine higher than 1.5mg/dL and stages 2 and 3, groups with higher mortality rates. The main advantage of this approach is that it allows for early treatment of patients with type one hepatorenal syndrome, henceforth HRS-AKI, without a need for establishing a threshold of 2.5mg/dL for diagnosis. Of the 13 nonresponders to our work, 9, that is 69%, did not fit previous criteria for type one hepatorenal syndrome since creatinine was below aforementioned value.

Child-Pugh score, in its turn, is a known prognostic indicator for cirrhotic patients. Although it does not include a marker for renal function, the score is directly proportional to blood levels of angiotensin and aldosterone, cardiac output, and portal venal pressure gradient, which are objective measures of cirrhotic circulatory disfunction, and also inversely correlate to renal perfusion [22, 23]. Scott et al. [16] found that mortality of cirrhotic patients with AKI was correlated to hepatic function according to Child-Pugh classification (3.1% stage A, 23.6% stage B, and 32.8% stage C; p=0.006), and stages B and C are independent factors associated with mortality. In a systematic review including 118 studies, Child-Pugh score was the most frequent independent predictor of mortality [21].

To our knowledge, this was the first prospective Brazilian study to evaluate prognosis of AKI in cirrhosis as well as association of AKI mortality and absence of response to expansion treatment, according to latest ICA consensus [12]. Findings confirmed dire prognosis for AKI in cirrhotic patients, both short and medium-term, according to the recently established ICA-AKI criteria. The main predictor for mortality was absence of response to volume expansion treatment. New studies are needed to investigate the impact of the new dynamic AKI definition establishment of early treatment for improved outcome of these patients.

## Figures and Tables

**Table 1:** Classification and definitions of AKI according to ICA-AKI.

**ICA-AKI Stages**	**Serum Creatinine**

Stage 1	Increase above 0.3mg/dL or elevation of 1.5 to 2 times baseline value

Stage 2	Increase between twice and three times baseline value

Stage 3	Increase greater than three times baseline value or of over 4mg/dL after an increase of at least 0.5mg/dL or need for dialysis

**Progression:** moving to greater stage or need for dialysis	
**Regression:** moving into milder stage	
**Response to treatment:**	
Complete: return to creatinine value under 0.3mg/dL of baseline	
Incomplete: regression in ICA-AKI stage or reduction of creatinine value of 0.3mg/dL or more of baseline value	
Absent: no regression of AKI.	

ICA = International Club of Ascites;

AKI = Acute Kidney Injury.

**Table 2:** Clinical and laboratory screening of 52 cirrhotic patients with AKI.

Characteristics	n (%)
(n=52)	

Child-Pugh Score (Average)	10,1(±2,2)
Child-Pugh Classification (A/B/C)	1(1,9%)/19(36,5%/32(62,5%)
MELD (Average)	21,9(±7,0)
MELD-Na (Average)	24,5(±6,7)
ICA-AKI Stages	
1	30(57,7%)
2	16 (30,8%)
3	6(11,5%)
Causes of AKI	
Infection	22(42,3%)
Hypovolemia	15(28,8%)
Hepatorenal syndrome	5(9,6%)
Nephropathy	3(5,8%)
Miscellaneous	3(5,8%)
Multiple	4(7,7%)
Creatinine (mg/dL) (Average)	2,12 (±0,9)
Total bilirrubin (mg/dL) (Average)	3,15(0,3-23,9)
Albumin (g/dL) (Average)	2,5(±0,4)
INR (Average)	1,7(1,0-6,9)
Sodium (mEq/L) (Average)	133,8 (±5,1)
C-Reactive Protein (mg/L) (Average)	10,1(±2,2)

ICA-AKI, International Club of Ascites–Acute Kidney Injury.

**Table 3:** Comparison of demographic, clinical, and laboratory screening variables at the end of the first month.

	Survivors (n=35)	Nonsurvivors (n = 14)	p value

**Demographic**			
** **Age	54.6 (±11.6)	55 (±8.7)	0.915
** **Men	24 (68.6%)	10 (71.4%)	0.845
** **Women	11 (31.4%)	4 (28.6%)	
**Clinical**			
** **Child-Pugh	9.9(±2.1)	11.3(±1.5)	0.019
** **MELD	20.4 (±6.0)	25.1 (±8.7)	0.034
** **MELD-Na	23.1 (±6.4)	27.6 (±7.3)	0.037
ICA-AKI Stage			0.269
** **1	20 (57.1%)	8 (57.1%)	
** **2	10 (28.6%)	6 (42.9%)	
** **3	5 (14.3%)	0	
Subdivision of stage 1 (n=28)			0.791
** **1a	6 (30%)	2 (25%)	
** **1b	14 (70%)	6 (75:%)	
AKI causes			0.609
** **Infection	14 (40%)	7 (50%)	
** **Hypovolemia	11 (31.4%)	4 (28.6%)	
** **Hepatorenal syndrome	2 (5.7%)	2 (14.3%)	
** **Nephropathy	2 (5.7%)	0	
** **Miscellaneous	2 (5.7%)	1 (7.1%)	
** **Multiple	4 (11.4%)	0	
Response to expansion treatment			
** **Complete	24 (68.6%)	4 (28.6%)	0.001
** **Partial	7 (20%)	1 (7.1%)	
** **Absent	4 (11.4%)	9 (64.3%)	
Encephalopathy			
** **Present	10 (28.6%)	4 (28.6%)	1.0
** **Absent	25 (71.4%)	10 (71.4%)	
Ascites			
** **Present	29 (82.9%)	12 (92.9%)	0.366
** **Absent	6 (17.1%)	1 (7.1%)	
**Laboratory**			
** **Creatinine (mg/dL)	2.2(±1.0)	1.8(±0.6)	0.165
** **Total bilirrubin (mg/dL)	1.72 (0.3-19.4)	6 (1.1-23.9)	0.028
** **Albumin (g/dL)	2.5 (±0.5)	2.4 (±0.5)	0.567
** **INR	1.5 (1.0-3.0)	2.0 (1.1-6.0)	0.012
** **Sodium (mEq/L)	133.2 (±5.4)	134.8 (±4.3)	0.329
** **C-Reactive Protein (mg/L)	30 (5-287)	31(13-197)	0.748

ICA-AKI, International Club of Ascites–Acute Kidney Injury.

**Table 4:** Comparison of demographic, clinical, and laboratory screening variables at the end of three months.

	Survivors (n =26)	Nonsurvivors (n = 22)	p value

**Demographic**			
Age	55.3 (±12.1)	54.1 (±9.5)	0.726
Men	17 (65.4%)	16 (72.7%)	0.584
Women	9 (34.6%)	6 (33.3%)	
**Clinical**			
Child-Pugh	9.7(±2.1)	10.9(±1.8)	0.046
MELD	19.3 (±6.1)	24.6 (±7.6)	0.012
MELD-Na	21.7 (±6.6)	27.5 (± 6.2)	0.03
ICA-AKI Stage			
1	17 (65.4%)	10 (56.3%)	0.259
2	6 (23.1%)	10 (45.5%)	
3	3 (11.5%)	2 (9.1%)	
Subdivision of stage 1 (n=28)			
1a	5 (29.4%)	2 (20%)	0.678
1b	12 (70.6%)	8 (80%)	
AKI causes			
Infection	10 (38.5%)	10 (45.5%)	0.058
Hypovolemia	9 (34.6%)	6 (27.3%)	
Hepatorenal syndrome	0	4 (18.2%)	
Nephropathy	2 (7.7%)	0	
Miscellaneous	1 (3.8%)	2 (9.1%)	
Multiple	4 (15.4%)	0	
Response to expansion treatment			<0.0001
Complete	20 (76.9%)	7 (31.8%)	
Partial	5 (19.2%)	3 (13.6%)	
Absent	1 (3.8%)	12 (54.5%)	
Encephalopathy			
Present	6 (23.1%)	7 (31.8%)	0.361
Absent	20 (76.9%)	15 (68.2%)	
Ascites			
Present	21 (80.8%)	12 (90.9%)	0.532
Absent	5 (19.2%)	2 (9.1%)	
**Laboratory**			
Creatinine (mg/dL)	2.0(±0.9)	2.2(±2.2)	0.525
Total bilirrubin (mg/dL)	1.6 (0.3-15.2)	5 (0.4-23.9)	0.020
Albumin (g/dL)	2.4 (±0.5)	2.6 (±0.5)	0.368
INR	1.5(1-3)	1.9(1.1-7)	0.067
Sodium (mEq/L)	134.1 (±5.2)	133.4 (±5.2)	0.681
C-Reactive Protein (mg/L)	30.4 (5-278)	31.6(5-278)	0.869

ICA-AKI, International Club of Ascites–Acute Kidney Injury.

**Table 5:** Analysis of Cox regression–final model (one and three months).

Time	One Month			Three Months		
	Odds Ratio	95% CI	P value	Odds Ratio	95% CI	P value

Response to treatment	0.26	0.13-0.52	<0.0001	0.25	0.14-0.45	<0.0001
Child-Pugh Score	1.63	1.2-2.21	0.001	1.49	1.18-1.89	0.001

CI: confidence interval.

## Data Availability

The data used to support the findings of this study are available from the corresponding author upon request.
